# The role of flagella and chemotaxis genes in host pathogen interaction of the host adapted *Salmonella enterica* serovar Dublin compared to the broad host range serovar *S.* Typhimurium

**DOI:** 10.1186/1471-2180-13-67

**Published:** 2013-03-25

**Authors:** John Elmerdahl Olsen, Kirsten Hobolt Hoegh-Andersen, Josep Casadesús, Jesper Rosenkranzt, Mark Simon Chadfield, Line Elnif Thomsen

**Affiliations:** 1 Department of Veterinary Disease Biology, Faculty of Health and Medical Sciences, University of Copenhagen, Stigbøjlen 4, Frederiksberg C, 1870, Denmark; 2 Department of Genetics, Faculty of Biology, University of Seville, Apartado 1095, Sevilla, 41080, Spain; 3 Novo Nordic, Krogshøjvej 51, Bagsværd, 2880, Denmark

## Abstract

**Background:**

The importance of flagella and chemotaxis genes in host pathogen interaction in *Salmonella enterica* is mainly based on studies of the broad host range serovar, *S.* Typhimurium, while little is known on the importance in host specific and host adapted serovars, such as *S.* Dublin. In the current study we have used previously characterized insertion mutants in flagella and chemotaxis genes to investigate this and possible differences in the importance between the two serovars.

**Results:**

*fliC* (encoding the structural protein of the flagella) was essential for adhesion and *fliC* and *cheB* (CheB restores the chemotaxis system to pre-stimulus conformation) were essential for invasion of *S.* Dublin into epithelial Int407 cells. In *S.* Typhimurium, both lack of flagella (*fliC/fljB* double mutant) and *cheB* influenced adhesion, and invasion was influenced by lack of both *cheA* (the histidine-kinase of the chemotaxis system), *fliC/fljB* and *cheB* mutation. Uptake in J774A.1 macrophage cells was significantly reduced in *cheA, cheB* and *fliC* mutants of *S.* Dublin, while *cheA* was dispensable in *S.* Typhimurium. Removal of flagella in both serotypes caused an increased ability to propagate intracellular in J774 macrophage cells and decreased cytotoxicity toward these cells. Flagella and chemotaxis genes were found not to influence the oxidative response. The induction of IL-6 from J774A-1 cells depended on the presence of flagella in *S.* Typhimurium, whilst this was not the case following challenge with *S.* Dublin. Addition of *fliC* from *S.* Typhimurium *in trans* to a *fliC* mutant of *S.* Dublin increased cytotoxicity but it did not increase the IL-6 production. Flagella were demonstrated to contribute to the outcome of infection following oral challenge of mice in *S. Dublin*, while an *S.* Typhimurium *fliC/fljB* mutant showed increased virulence following intra peritoneal challenge.

**Conclusions:**

The results showed that flagella and chemotaxis genes differed in their role in host pathogen interaction between *S.* Dublin and *S.* Typhimurium. Notably, lack of flagella conferred a more virulent phenotype in *S.* Typhimurium at systemic sites, while this was not the case in *S.* Dublin. *In vitro* assays suggested that this could be related to flagella-induced induction of the IL-6 pro-inflammatory response, but further *in vivo* studies are needed to confirm this.

## Background

The flagellum of *Salmonella enterica* is made up of a single protein, flagellin, which consists of approximately 490 amino acids, and which differs between serovars [1]. For example *fliC* of *S.* Dublin and *S.* Typhimurium shows 38 % identity at the DNA-level (BLASTN 2.2.1, NCBI) and 54 % identity at the amino acid level. *Salmonella* consist of more than 2500 serovars, most of which have two flagellin genes, *fliC* and *fljB*, allowing antigen alteration [2]. The latter has been lost by secondary deletion in some lineages [3], for example *S.* Dublin only expresses flagellin encoded by *fliC*. A recent review suggests an evolutionary model, where *fliC* is the original and preferred gene, and *fljB* is only used under particular environmental conditions [3]. Flagella confer the ability of the bacterium to swim in liquid media. Chemical information received at membrane-receptors influence the rotation of the flagellum motor, thus enabling the bacteria to respond to changes in the external environment by ordered motility. This signal transduction happens through the chemotaxis system (reviewed by Kojima and Blair [4]).

Flagella are recognized as PAMPs (pathogen associated molecular patterns) used by the host to recognize bacteria and besides their function in motility, flagella of *S.* Typhimurium have been shown to stimulate both the innate and adaptive immune system. Extracellular flagella activate toll-like receptor 5 (TLR-5) leading to a pro-inflammatory response with induction of cytokines (reviewed by Kawai and Akira [5]). Soluble flagellin in the cytosol induces pyroptotic cell death (see review by Miao *et al.*[6]) in a caspase-1-dependent manner through activation of the Nod like receptor NLRC4. This is in particular relevant in relation to intracellular bacteria, such as *Salmonella*, and a strain of *S.* Typhimurium that was manipulated to be unable to down regulate *fliC* expression intracellular was demonstrated to be attenuated during systemic infection [7].

Conflicting results have been reported on the importance of chemotaxis, flagellation and motility in host pathogen interaction in *Salmonella.* Flagella were found to be important for *S.* Typhimurium invasion of MODE-K and Henle-407 cells, also when centrifugation was applied to maximize bacteria-to-cell contact. Hence the effect was considered unrelated to motility [8]. At the same time point, mutation of *fliC* and mutation of the motor protein *motA* did not to influence intracellular cell numbers of *S.* Enteritidis in CaCo-2 cells [9]. This may, however, be a strain or cell specific response, since mutants of another *S.* Enteritidis strain showed reduced invasion in both Hep-2 and Div-1 cells [10]. Early challenge experiments using C57BL/6J mice reported that flagella, whether functional or not, were virulence factors in *S.* Typhimurium, while chemotaxis genes were dispensable [11]. However, subsequent studies, with other strains have not been able to confirm the flagella phenotype [8,12]. Flagella but not fimbriae and not motility were found to be essential for *S.* Enteritidis infections in chicken [13], and lack of flagella causes a disadvantage in the early stage of oral infection of rats and in cell culture invasion [14,15].

*Salmonella* serovars have very different epidemiology and life style, just as they display obvious differences with regard to motility and chemotaxis. The commonly studied *S.* Typhimurium infects numerous hosts and displays phase variation of its flagella antigens. The host-specific and host-adapted serovars, on the other hand, infect a single or few hosts, and do not rely on extra-animal survival to any great extend [16]. It may be that motility and chemotaxis play a different role during host pathogen interaction in different serovars, depending on their lifestyle.

The current understanding of the importance of flagella and chemotaxis genes in *Salmonella* host pathogen interaction is derived from studies of *S.* Typhimurium and *S.* Enteritidis, and results based on these serovars are taken as general for the genus. Since the lifestyle differs markedly between ubiquitous serovars and the host-specific/host-adapted ones, we hypothesized that this may be a wrong assumption. In order to investigate this, we characterized the importance of chemotaxis and flagella genes for host pathogen interaction of the host-adapted serovar *S.* Dublin compared to the well-characterized serovar *S.* Typhimurium.

## Results

### Interaction with epithelial cells

*Salmonella* normally infects through the faecal oral route. Several studies have reported that flagella are important for the intestinal phase of infection, mostly based on studies of the initial contact between cultured cells and flagella and motility mutants [8,17]. In this study we compared the adhesion and invasion of a wild type strain of *S.* Dublin to the smooth swimming *cheA* mutant, the tumbling *cheB* mutant and a mutant without flagella (*fliC* mutant). The corresponding mutants of *S.* Typhimurium were used as reference points. The results are shown in Table 1. The *S.* Dublin flagella mutant (*fliC*) was significantly reduced in adhesion and invasion, the constitutively tumbling *cheB* mutant was reduced in invasion, while the constitutively smooth swimming (*cheA* mutation) only showed a slight, non-significant reduction of adhesion and invasion. As can be seen from the Table 1, the flagella phenotype paralleled that of the flagella-less *S.* Typhimurium mutant, while *cheA*-mutation caused significantly reduced invasion and *cheB-*mutation both reduced adhesion and invasion in this serotype.

**Table 1:** **Adhesion and invasion of *S. *Dublin (SDu) and *S. *Typhimurium (STm) WT and flagella and chemotaxis mutants**^**a **^**in cultured epithelial Int407 cells**

**Strain**	**Adhesion (% of wild type)**	**Invasion (% of wild type)**
SDu *cheA*	47.8 ± 5.6	83.4 ± 8.0
SDu *cheB*	19.5 ± 7.8	2.4 ± 0.9***
SDu *fliC*	6.0 ± 3.3***	1.0 ± 0.3***
STm *cheA*	76.2 ± 33.5	40.8 ± 10.9^**^
STm *cheB*	15.6 ± 2.7***	1.2 ± 1.3***
STm *fliC/fljB*	12.5 ± 1.9***	0.4 ± 0.3***

a: Performance of mutant strains was compared statistically to the wild type strain of the same serovar. **: p<0.01; ***: p<0.001.

The inoculum of each strain was between Log_10_ 7.9 and Log_10_ 8.2 with no significant difference between strains.

### Uptake and survival inside macrophages

Once *Salmonella* has invaded the host, professional phagocytic cells quickly take up the bacteria. Especially the uptake by macrophages has been considered important, deduced from the fact that all *S.* Typhimurium mutants that are attenuated for macrophage survival have turned out to be non-virulent in challenge experiments [18]. To investigate whether macrophage interaction depended on the presence of flagella and chemotaxis genes, we conducted experiments with cultured J774A.1 cells. The results are shown in Table 2. *S.* Dublin strains with mutation in *cheA, cheB* and *fliC* were taken up by macrophages in significantly lower numbers than the wild type strain. The mutants of *S.* Typhimurium were found to have the same general uptake phenotypes, however, the differences between the wild type strain and the *cheA* mutant were not significant. All strains increased in numbers from 3 to 24 hours, but due to relatively large standard deviations, only the difference in net growth of the *S.* Typhimurium *fliC/fljB* mutant was statistically different from that of the wild type strain. At 48 hours, wild type and chemotaxis mutants decreased in numbers, however, the *cheB* mutant of *S.* Typhimurium was significantly less reduced compared to the wild type strain. Contrary to this, flagella-less mutants of both serotypes showed net growth, but only the *S.* Typhimurium strains was significantly different from the wild type strains.

**Table 2:** **Uptake and survival of *S. *Dublin 3246 (SDu) and *S. *Typhimurium (STm) wildtype and flagella and chemotaxis mutants in cultured J774A.1 macrophages**^**a**^

**Strain**	**Uptake 3h (Percent of wild type strain)**	**Survival 24 h (Percent of same strain at 3h)**	**Survival 48 h (Percent of same strain at 3 h)**
SDu WT	100	124,1 ± 43.5	20.7 ± 4.7
SDu *cheA*	53.9 ± 15.1**	279.8 ± 65.8	53.8 ± 16.5
SDu *cheB*	1.4 ± 1.0**	307.7 ± 90.2	248.8 ± 39.8
SDu *flic*	1.0 ± 0.2***	450.5 ± 255.0	615.3 ± 325.8
STm WT	100	114.0 ± 42.6	2.8 ± 1.72.8
STm *cheA*	72.4 ± 22.4	100.2 ± 31.0	12.2. ± 3.1
STm *cheB*	19.0 ± 9.3**	309.8 ± 231.5	81.7 ± 6.9*
STm *fliC/flijB*	0.2 ± 0.1***	490.9 ± 111.6*	702.9 ± 53.0***

a: Uptake of mutant strains was expressed relatively to and compared statistically to the wild type strain of the same serovar. Survival at 24 and 48 hours was expressed relatively to the number of bacteria determined at 3 hours and compared statistically to the survival capability of the wild type strain of the same serotype. *: p<0.05; **: p<0.01 ***: p<0.001.

### Cytotoxicity towards macrophage cell line J774A.1

**Figure 1:**
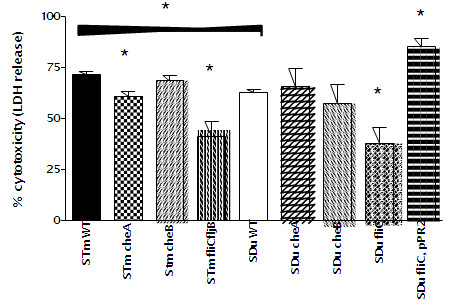
**Cytotoxicity of strains of *S. *Dublin (SDu) and *S. *Typhimurium (STm) in J774A.1 macrophages.** Cytotoxicity was measured 24 hours post challenge with flagellar (SDu *fliC* and STm *fliC/fljB*) and chemotaxis mutants (*cheA* and *cheB*) and the wild type strains. Significant (p<0.05) differences between wild type and mutant strains are shown with *. The cytotoxicity of the two wild type strains was also compared, and this was shown to be statistically different, as indicated by the * in the top of the figure.

Results of the macrophage assays above may be influenced by cytotoxicity of the strain, since strains that kill the macrophages subject themselves to the action of the antibiotic gentamicin in the culture medium. A comparison of cytotoxicity towards the J774A.1 cells after 24 hours is shown in Figure 1. The non-flagellated mutants of *S.* Dublin and *S.* Typhimurium were less cytotoxic than the wild type strains, in line with previous observations that flagella influence *Salmonella* induction of macrophage cell death [19]. The net growth of flagella mutants in the survival assays above could thus be a result of decreased killing of macrophages. The chemotaxis mutants of *S.* Dublin did not differ significantly from the wild type strain, while the *cheA* mutant of *S.* Typhimurium was slightly, but significantly, less cytotoxic than the wild type strain.

Wild type *S.* Dublin was less cytotoxic than wild type *S.* Typhimurium (Figure 1). To investigate whether this was related to the flagella type, we provided the *fliC* mutant of *S.* Dublin with *S.* Typhimurium *fliC in trans* on the plasmid pPR2. The *fliC* mutant itself was negative with H:*p,g* (*S.* Dublin flagella antigen) and H:*i*, H:*2* (*S.* Typhimurium flagella antigen) by serotyping and Western blot, while the complemented strain was positive with H:*i* and H:*2* typing sera. It was non-motile but expressed a high number of flagella as demonstrated by electron microscopy (data not shown). It did not differ significantly from the wild type strain in interactions with epithelial cells or macrophages (data not shown). The complemented *fliC* mutant of *S.* Dublin was significantly more cytotoxic than the wild type strain of *S.* Dublin, above the level of the wild type strain of *S.* Typhimurium (Figure 1).

### The importance of chemotaxis and flagella genes for induction of oxidative burst in macrophages

**Figure 2:**
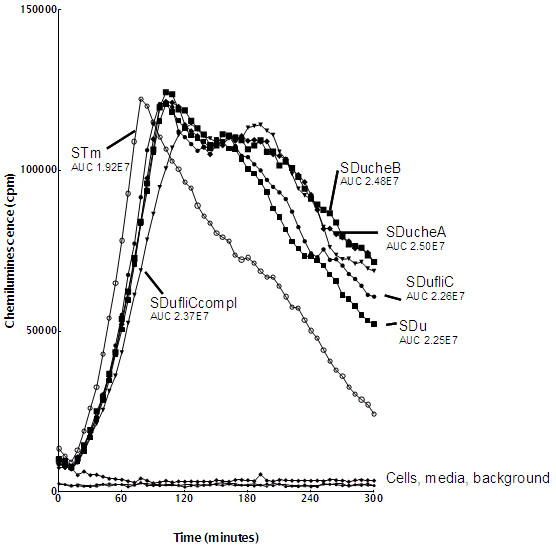
**Oxidative responses of J774A.1 macrophages following challenge with wild type and chemotaxis and flagella mutant of *S. *Dublin (SDu) and *S. *Typhimurium (STm).** The response is measured in arbitrary chemiluminescence units. Positive and negative controls are indicated.

The ability of the strains to stimulate the oxidative burst in J774A.1 cells was investigated. Wild type strains differed in induction of oxidative response in the sense that the wild type strain of *S.* Typhimurium peaked early compared to the wild type strain of *S*. Dublin, and showed a significantly lower area under the response curve (AUC). Only relative small differences in the oxidative burst were observed between *S.* Dublin wild type and mutant strains, and none of the differences were statistically significant (Figure 2). Knock out of flagella and chemotaxis genes also did not influence the oxidative burst significantly in *S.* Typhimurium (data not shown). When the *S.* Dublin *fliC* mutant was complemented with *S.* Typhimurium *fliC*, the response peaked later but the magnitude of response (AUC) was not affected (Figure 2).

### Induction of cytokines IL-6 response in cultured J774A.1 macrophages

**Figure 3:**
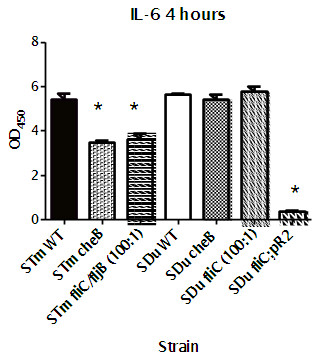
**Induction of IL-6 response in J774A.1 cells 4 hours post challenge with wild type and chemotaxis and flagella mutants of *S. *Dublin and *S. *Typhimurium.***cheA* mutants that had not given any phenotype in cell culture and mice assays were omitted from this analysis. As a control for level of uptake, the cells were challenged with flagella mutants of both serovars with MOIs of both 10:1 and 100:1. Results from the two testings were not significantly different. Only 100:1 results are shown in the figure. Significant (p<0.05) differences to the wild type strain of the same serovar are indicated by *.

As mentioned in the introduction, flagellin has been reported to stimulate a pro-inflammatory response with induction of cytokines including IL-6 [5]. We wanted to investigate how the IL-6 response depended on the presence of flagella and chemotaxis genes. After 1 hour, no significant IL-6 production was seen in any of the strains (data not shown), however, after 4 hours, strains of both serovars had induced a strong production of IL-6 (Figure 3). In *S.* Typhimurium, mutation in both flagella genes independently or together, as well as mutation of *cheB*, caused a reduced IL-6 response, while surprisingly, lack of flagella did not cause a reduction in *S.* Dublin. IL-6 levels following challenge of cells with ten times higher doses of *S.* Typhimurium *fliCfljB* and *S.* Dublin *fliC* mutants did not change the responses compared to the normal challenge dose. Complementation of *fliC* in *S.* Dublin with *fliC* from *S.* Typhimurium *in trans* caused a dramatic reduction of IL-6 from the infected macrophages.

### Oral and intra peritoneal challenge of mice

The chemotaxis mutants did not differ significantly from the wild type strains following oral challenge. The *S.* Dublin *fliC* mutant showed lower CFU in the spleen 4–5 days post challenge (CI: 0.46 (p<0.01)), while the *S.* Typhimurium *fliC/fljB* mutant did not differ markedly from the wild type strain (CI: 1.12), however, the difference was statistically significant. Lack of flagella has been reported to increase fitness of *S.* Typhimurium during systemic infection of mice [8]. We therefore also investigated the importance of flagella genes using intra peritoneal challenge, thereby bypassing the intestine. The *S.* Typhimurium *fliC/fljB* mutant showed increased numbers of bacteria in the spleen (CI: 1.78; p<0.0001), which corroborates previous studies [8]. The corresponding flagella-less *S.* Dublin mutant did not show this phenotype (CI: 0.91) (Table 3).

**Table 3:** **Virulence phenotypes of flagella and chemotaxis mutants of *S. *Dublin (SDu) and *S. *Typhimurium (STm) in C57/B6 mice**

**Mutant**	**Challenge route**^**a**^	**CI**^**b **^**S.Du**	**CI**^**b **^**STm**
*cheA*	p.o.	1.03	1.09
*cheB*	p.o.	0.97	1.05
*fliC*	p.o.	0.46^**^	-
*fliC*	i.p.	0.91	-
*fliC/fljB*	p.o.	-	1.12**
*fliC/fljB*	i.p.	-	1.78^***^

a: p.o. = per oral challenge; i.p. = intraperitoneal challenge.

b: The competitive index was calculated as the ratio of mutant to wild type in the spleen 4–5 days post infection divided by the ratio of mutants to wild type strain in the input pool. Indexes where the output was significantly different from the input pool are marked with ** (p<0.01) and *** (p<0.001).

## Discussion

In the current study we used chemotaxis and flagella mutants of the host adapted serovar *S.* Dublin and corresponding mutants of the broad host range serovar *S.* Typhimurium to study possible serovar differences in the importance of these genes for host pathogen interaction. The studies were based on defined mutants in one strain of each serovar, and we cannot rule out that there may be strain differences within serovar.

The constitutively tumbling *cheB S.* Dublin mutant, but not the constitutively smooth swimming *cheA* mutant, was negatively affected in invasion of epithelial cells. Since *cheA* has previously been shown to be important for *S.* Typhimurium cell invasion [20], which we also observed in our studies, *S.* Typhimurium and *S.* Dublin apparently differ with respect to the role of *cheA* in epithelial cell invasion. Lack of flagella (*fliC* mutation) caused reduced adhesion, which is in accordance with previously reported results for the effect of *fliC/fljB* mutation in *S.* Typhimurium [17] and our observations on the role of flagella in this serotype.

It has previously been reported that it is the flagella and not motility, which are important for cell adhesion and invasion [17], but it is currently unknown how precisely flagella influence this in a motility independent way, at least in cell culture experiments. Since we used centrifugation to maximize cell contact, it is also unlikely that our results were caused by reduced motility, which would lead to a reduction in number of contacts between bacteria and cells. Flagella in *S.* Typhimurium are expressed inside epithelial cells and can be demonstrated in infected cultured HeLa cells [21]. During *in vivo* invasion, the stimulation of TLR-5 by flagellin and the following pro-inflammatory response may be important. However, invasion by *S.* Typhimurium in cell culture experiments happens within 15 minutes [22], and it is unlikely to be influenced by secretion of stimulating factors. A more likely explanation is down-regulation of SPI1 in flagella mutants, as suggested by Kim *et al.*[23]. This down regulation can be caused by several regulatory systems, which control both flagella and virulence gene expression [24,25].

Whether motility *per se* can be ruled out as important for invasion is still controversial since motility was shown to be essential for invasion of *S.* Typhi into cultured epithelial cells [26]. A recent study with *S.* Typhimurium also suggests a requirement for motility in infection of epithelial cells. The invading population was demonstrated to consist of two populations. Some cells were only infected with few bacteria, which did not multiply to any great extent. These bacteria showed down-regulation of SPI-1 and *fliC* transcription. A fraction of approximately 10% of cells, however, was infected with bacteria that were motile, expressed invasion genes, possessed flagella, and multiplied at high rate. A speculation is that these cells may be ready to re-enter the lumen of the intestine to re-infect other cells [22]. Whether a similar picture can be seen for *S.* Dublin remains to be investigated.

Similar to invasion into epithelial cells, mutation of chemotaxis and flagella genes caused reduced uptake by macrophage cells. The reason for this is unknown. The flagella and chemotaxis genes are down regulated once *S.* Typhimurium is inside a macrophage [27], probably to prolong the time the bacterium can stay inside the macrophage protected from neutrophil killing in the extracellular environment [7]. The intracellular down regulation is controlled by the gene *ydiV,* which prevents transcription of the flagellin promoter [28]. It is currently unknown how *S.* Dublin regulates it flagella expression in response to macrophage uptake. Despite the down regulation, flagella of *S.* Typhimurium are important for the outcome of the systemic phase of an infection, since lack of flagella leads to a decrease in the percentage of CD14+ and CD54+ cells resulting in a reduction of uptake of soluble antigens by these cells and fewer bacteria accumulating intracellular [29,30]. Flagellin induces I-κBα degradation and subsequent NF-κB nuclear translocation, and induction of nitric oxide synthase [31-33]. This induces rapid *de novo* synthesis of tumour necrosis factor alpha (TNF-α), interferon gamma (IFN-γ), interleukin-1β (IL-1β) followed by IL-6 and IL-10, which is typical for a systemic inflammatory response.

Lack of flagella was found to allow net growth inside the macrophages over a 48 hours period, while wild type and chemotaxis mutant strains were reduced in numbers. The SPI-1 encoded type three-secretion system and flagella are important for rapid host cell death by pyroptosis seen after cell infection with *S.* Typhimurium [19]. In the present investigation, lack of flagella caused reduced extracellular levels of lactate dehydrogenase, the intracellular enzyme used as an indicator of macrophage cell death, and this reduced killing can be the reason for the net growth observed with flagella-less mutants. The present investigation does not allow us to conclude which underlying mechanism that was responsible for the reduced cell death when flagella were absent. Wild type *S.* Typhimurium was significantly more cytotoxic than *S.* Dublin. When *S.* Dublin expressed *S.* Typhimurium *fliC,* the cytotoxicity increased above *S.* Typhimurium levels. This indicates that *fliC* is important for the level of cytotoxicity, however, the complemented strain used to show this had a higher number of flagella than the wild type strain, and we cannot rule out that this causes the increase in cytotoxicity. The plasmid used for complementation was based on pMF3, which has previously been used to complement knock out phenotypes in *S.* Typhimurium without adverse effects [34]. More detailed studies are needed to demonstrate how these serotype differences relate to differences in the flagella sequence.

Significant cytokine production is generally assumed to require phagocytosis of the bacteria [35]. This corresponds to uptake in our assays, and as pointed out by Winther *et al.*[36] knock out mutants are not well suited to distinguish between lack-of-stimulation and lack-of-internalization responses. The flagella mutant of *S.* Typhimurium caused a reduced IL-6 cytokine production, but it also showed reduced uptake. We therefore included a control experiment where a 10 times higher challenge dose of the flagella mutant was used. The high challenge dose did not increase the IL-6 production, indicating that the lack of response was most likely not related to invasion levels. In support of this conclusion, the *fliC* and *cheB* mutants of *S.* Dublin also showed significantly reduced invasion, but absence of these genes in *S.* Dublin did not influence cytokine production. This result point to a fundamental difference between *S.* Dublin and *S.* Typhimurium in the way the flagella stimulates the host response, and calls for more detailed studies on structural functional relations in the signalling to the host.

The *S.* Dublin *fliC* mutant with *S.* Typhimurium provided *in trans* induced a lower response than the wild type strain. This result was surprising. Its phenotype is similar to a *motA* mutation, i.e. structurally the flagella appears normal, but they do not move. Naturally occurring *motA* mutants of *S.* Enteritidis stimulated transcriptional pro-inflammatory responses in Caco-2 cells [37], and there is no obvious reason why the complemented *S.* Dublin strain should not do the same. In cell culture experiment, a *motA* mutant of *S.* Typhimurium was non-invasive [19], which differs from the phenotype of our complemented mutant, and further studies are needed to clarify this observation.

Lack of stimulation of IL-6 expression has previously been seen with the host-specific serovar *S.* Gallinarum in a comparison to *S.* Typhimurium and *S.* Enteritidis after infection of a primary chicken cell line [38]. No control was included in that study for the fact that *S.* Gallinarum contrary to *S.* Typhimurium and *S.* Enteritidis lacks flagella. Our results indicate that lack of IL-6 induction may be a general feature of host adapted/ host specific serotypes. Host specific serovar has been speculated to perform stealth like infection (i.e. down regulates several host responses) in comparison to the ubiquitous serovars [39]. The lower cytotoxicity and lack of IL-6 responses support this assumption. In contrast to the role in IL-6 induction, none of the mutants differed significantly from the wild type strains in induction of oxidative responses. This result suggested that flagellin was not important for induction of the oxidative response.

Results on the role of flagella and chemotaxis genes in *Salmonella* host pathogen interaction have been contradictory (compare [12] and [8] with [11]), and we purposely looked for a sensitive assay to show subtle differences between strains. Co-infection assays have been shown to be more sensitive than assays where strains are tested individually [40]. Using this assay, we found that flagella significantly influenced the number of bacteria that could be isolated from the spleen 4–5 days post oral infection of mice with *S.* Dublin, but not with *S.* Typhimurium. Chemotaxis genes were found to be dispensable in this assay, as previously reported for *S.* Typhimurium [11]. Animal welfare regulations dictated us to scarify mice when they were severely affected by infection, and this prevented us from using one single end-point of infection. Potentially, this may have influenced the competitive indexes for *S.* Typhimurium, since this serovar propagated at different speed at systemic sites depending on the presence of flagella genes (see below). However, all mice were killed within a 24 hours period, and we do not believe that this significantly influenced our results.

Like *cheA* mutation, mutation of *cheR* confers a constitutively smooth swimming phenotype. We have not included this gene in our investigation, and we cannot rule out that it may have a different role in host pathogen interaction than *cheA*. We have performed preliminary testing of an *S.* Dublin *cheR* mutant and found that it corresponds to *cheA* with respect to phenotypes in cell assays and oral challenge of mice (unpublished), however, we do not have *S.* Typhimurium results to compare it to.

Flagella have been found to be important for the outcome of oral infection with *S.* Typhimurium in streptomycin treated mice, which is a model for studies of the entero-pahtogenesis of *Salmonella*[41]. In this model flagella are essential for initiation of inflammation, creating an environment in which *Salmonella* prevails over the normal flora, and in this model, chemotaxis genes were also essential for the outcome of infection. Cattle are the natural host for *S.* Dublin, and in addition to differences caused by the choice of animal model, studies have shown that virulence factors may differ depending on the host [42]. This must be taken into account when concluding on the current results. The changes in virulence observed when flagella were removed were relatively modest. We have previously demonstrated that flagella do not play an important role during extra animal survival [43], and we believe that selection for stable maintenance of the flagella apparatus must happen during the interaction with the host. It thus appears that these small differences are enough to provide the selective force.

It has previously been reported that a flagella mutant of *S.* Typhimurium is hyper virulent following intraperitoneal challenge of mice [8] and we confirmed this result. In contrast, the *S.* Dublin flagella mutant was not different from the wild type strain after intraperitoneal challenge. In conjunction with the results of IL-6 induction and cytotoxicity, this indicates that flagella are most important for *S.* Dublin in the initial invasion phase in the intestine, while it plays a minor role during the systemic phase. We suggest that a likely explanation for the contradicting results on the role of flagella in virulence of *S.* Typhimurium is that the results depends very much on the time point where bacterial load is measured. At early time points, lack of flagella causes a lower invasion, but at later time points, this is balanced by a higher ability to grow in the systemic phase.

## Conclusion

The results show that flagella but not chemotaxis genes influence the outcome of *S.* Dublin infection following oral challenge in the mouse model, and that *S.* Dublin flagella do not appear to be important during the systemic phase of infection. This points to fundamental differences in bacteria host signalling between *Salmonella* serotypes, and shows that results from studies of *S.* Typhimurium cannot be assumed to be general to the genus.

## Methods

### Strains and growth conditions

Well characterized flagella and chemotaxis insertion mutants of *S.* Dublin 3246 and *S.* Typhimurium 4/74 (Table 4) were obtained from a previous study [43]. The pMF3 derived plasmid pPR2 (TH2422) encoding *S.* Typhimurium *fliC* was kindly provided by Dr. Kelly T. Hughes, Washington University, Seattle, USA and was used to provide this gene *in trans* to *S.* Dublin. Plasmid extraction was performed with the QIAgen purification kit, as described by the manufacturer and electroporation was carried out as described by Maloy *et al*. [44].

**Table 4:** Bacterial strains and their motility phenotypes

**Strain**	**Description; Relevant genotype**	**Motility phenotype**	**Source**
JEO 3774	Wild-type *Salmonella* Typhimurium 4/74	Wild type	[45]
JEO 3665	Wild-type *Salmonella* Dublin 3246	Wild type	[45]
JEO880	JEO 3774 (*cheA*::Tn10^a^)	Smooth	[43]
JEO881	JEO 3774 (*cheB*::Tn10^a^)	Tumbling	[43]
JEO885	JEO 3774 (*fliC*::Mu*d*J; *fljB*::Mu*d*JCm^e^)	None	[43]
JEO886	JEO 3665 (*fliC*::Mu*d*J^b^)	None	[43]
JEO887	JEO 3665 (*fliC*::Mu*d*J; pPR2^d^)	None	This study
JEO888	JEO 3665 (*cheA*::Tn10^a^)	Smooth	[43]
JEO889	JEO 3665 (*cheB*::Tn10^a^)	Tumbling	[43]

^a^ Tet^r^; ^b^ Kan^r^; ^c^ Chloram^r^; ^d^ Kan^r^,Amp^r^; ^e^ Kan^r^,Chloram.^r^

Unless otherwise stated, strains were cultured in LB broth (Difco) overnight at 37°C. Stock cultures were maintained frozen at −80°C in LB supplemented with glycerol (33 % w/v). To prepare inoculate for *in vivo* and *in vitro* studies, stocks were thawed and inoculated on LB-agar containing 1,5% agar (Difco) and plates were incubated overnight at 37°C. Single representative colonies were inoculated into fresh LB broth and incubated overnight at 37°C. Media were supplemented with relevant antibiotics (Sigma) at concentrations: kanamycin (50 *μ*g/ml), tetracycline (20 *μ*g/ml), ampicillin (50 *μ*g/ml) and chloramphenicol (20 *μ*g/ml).

### Motility measurement

Motility was assayed in Heart Infusion broth with 0.25 % agar (Difco) and on Swarm agar (Statens Serum Institute, DK) as described [43].

### Expression of flagella antigens

Serotyping was performed as previously described [43]. Western blot was performed using NuPAGE™ 12,5% Tris–HCl gels (Novex) as instructed by the manufacturer and specific flagella antisera (H:i, H:2 or H:p,g), (Statens Serum Institute (SSI), Denmark).

### Demonstration of flagella by electron microscopy

To demonstrate flagella, bacteria were negatively stained with uranyl acetate 2% and examined by transmission electron microscopy at an instrumental magnification of 27500.

### Adhesion and survival properties *in vitro*

Comparison of *in vitro* adhesion, invasion (uptake) and survival of bacteria inside cells was performed using the epithelial cell line Int407 and the macrophage-like cell line, J774A.1, as previously described [46]. Before experiments with macrophages, bacteria were opsonised with 10 % heat treated foetal calf serum (Invitrogen) for 30 min at 37°C prior to addition to the cells. Infections were performed at a multiplicity of infection (m.o.i.) of 10:1 with the macrophage cell line and 100:1 with Int407 cells. For all experiments, cells were centrifuged at 1500 rpm for 2 min. immediately after infection to allow close contact of the bacteria with the cells. Each bacterial strain was assayed in triplicate and experiments were repeated once.

### Cytotoxicity

Cytotoxicity to macrophages was determined by release of lactate dehydrogenase (LDH) by the monolayers into supernatants using the CytoTox 96® Non-Radioactive Cytotoxicity Assay (Promega G1780). Results were expressed as the percentage of LDH released by infected monolayers compared to LDH release by lysis buffer treated (lysed) monolayers at 24 hours [(A_495_ test sample – A_495_ medium control) / (A_495_ macrophage+lysis buffer – A_495_ medium+lysis buffer)] × 100.

### Induction of oxidative radicals (chemiluminescence)

The method described by Chadfield and Olsen [47] was used. Opsonized zymosan (Sigma) and phorbol myristate acetate (PMA)(Sigma) was used as positive control stimuli. A Lucigenin probe (Sigma) dissolved in DMSO (Sigma) and diluted in Hanks balanced salt solution (HBBS) (Gibco Life Technologies) to final assay concentrations of 150 μg/ml was used. Cells used in the assays were J774A.1. The luminometer (AUTOLUMAT LB 953, Berthold) was set at 37°C. The reading intervals were minutes and the duration of the assays were 300 minutes. The response was expressed as the area under the response curve (AUC) and the time where the response peaked.

### Induction of IL-6 production

A macrophage invasion assay was conducted with J774A.1. After 1 hour and 4 hours of incubation, the last 3 hours with gentamicin present in the medium, supernatants were removed and assayed for the presence of cytokine IL-6 using a commercially available kit (Promokine, mouse IL-6 ELISA kit). Positive controls consisted of purified IL-6 supplied with the kit, and negative controls consisted of wells not infected with bacteria.

### Animal challenge experiments

Per oral and intraperitoneal virulence were assessed using competitive challenge assays with five C57BL/6 female mice (Taconic Black6 mice) of 6–8 weeks of age per group. The protocol followed the instructions of Jelsbak *et al.*[48] for intra peritoneal challenge, while a challenge dose of 8 × 10^6^ CFU was used for per oral challenges. In all experiments, *S.* Dublin was given a 10 times reduced dose compared to *S.* Typhimurium. The ratio between the wild type and the mutant strain in the broth used for challenge as well as the ratio in the spleen 4–5 days post challenge was determined by patching of 100 colonies from the broth and from the spleen of each mice onto LB agar without antibiotic and 100 colonies onto LB agar with the relevant antibiotic. For statistical analysis of the difference between input and output ratios, an estimate of the variation on the input ratio was needed. This was obtained by combining the results from the patching of all input pools into one distribution and using this as an average input ratio. The animal experimentation was conducted with permission from the Animal Experiments Inspectorate (http://www.foedevarestyrelsen.dk/Dyr/Dyrevelfaerd/Dyreforsoegstilsynet/Sider/forside.aspx) in accordance with Danish law (license number: 2009/561–1675).

### Statistical analysis

Statistical analyses were made using the statistical software package GraphPath Prism 5. Mean CFU of bacterial strains in cell assays and cytotoxicity levels were compared using Bonferroni’s multiple comparison test. Comparison of mean competitive index between wild type and mutant strains and oxidative responses were done using unpaired T-test. P<0.05 was considered significant.

## Abbreviations

CI: Competitive index; AUC: Area under curve; CFU: Colony forming units

## Competing interests

The authors declare that they have no competing interests.

## Authors’ contributions

KH-H-A and JC constructed the strains, KH-HA, MSC and JEO conducted cell culture adhesion and invasion experiments, MSC measured the oxidative response in macrophages, JEO measured cytokine responses, KH-HA, JEO and JR conducted mice challenge experiments, JEO drafted the manuscript and all authors read and commented on this. All authors approved the final manuscript.
